# Proteomic and Low-Polar Metabolite Profiling Reveal Unique Dynamics in Fatty Acid Metabolism during Flower and Berry Development of Table Grapes

**DOI:** 10.3390/ijms242015360

**Published:** 2023-10-19

**Authors:** Patricio Olmedo, Juan Vidal, Excequel Ponce, Bruno G. Defilippi, Alonso G. Pérez-Donoso, Claudio Meneses, Sebastien Carpentier, Romina Pedreschi, Reinaldo Campos-Vargas

**Affiliations:** 1 Escuela de Agronomía, Facultad de Ciencias Agronómicas y de los Alimentos, Pontificia Universidad Católica de Valparaíso, Quillota 2260000, Chile; patricio.olmedo@pucv.cl (P.O.); juan.vidal@pucv.cl (J.V.); excequel.ponce@pucv.cl (E.P.); 2 Unidad de Postcosecha, Instituto de Investigaciones Agropecuarias (INIA) La Platina, Santiago 8831314, Chile; bdefilip@inia.cl; 3 Departamento de Fruticultura y Enología, Facultad de Agronomía y Sistemas Naturales, Pontificia Universidad Católica de Chile, Santiago 7820436, Chile; agperez@uc.cl (A.G.P.-D.); claudio.meneses@uc.cl (C.M.); 4 Facultad de Ciencias Biológicas, Pontificia Universidad Católica de Chile, Santiago 8331150, Chile; 5 Millennium Nucleus for the Development of Super Adaptable Plants (MN-SAP), Santiago 8370186, Chile; 6 Millennium Institute Center for Genome Regulation (CRG), Santiago 7800003, Chile; 7 Facility for Systems Biology Based Mass Spectrometry SYBIOMA, KU Leuven, B-3000 Leuven, Belgium; sebastien.carpentier@kuleuven.be; 8 Bioversity International, Biodiversity for Food & Agriculture, B-3001 Leuven, Belgium; 9 Centro de Estudios Postcosecha, Facultad de Ciencias Agronómicas, Universidad de Chile, Santiago 8831314, Chile; reinaldocampos@uchile.cl

**Keywords:** *Vitis vinifera*, proteomics, metabolomics, triacylglycerol, lipid, β-oxidation

## Abstract

Grapevine development and ripening are complex processes that involve several biochemical pathways, including fatty acid and lipid metabolism. Fatty acids are essential components of lipids, which play crucial roles in fruit maturation and flavor development. However, the dynamics of fatty acid metabolism in grape flowers and berries are poorly understood. In this study, we present those dynamics and investigate the mechanisms of fatty acid homeostasis on ‘Thompson Seedless’ berries using metabolomic and proteomic analyses. Low-polar metabolite profiling indicated a higher abundance of fatty acids at the pre-flowering and pre-veraison stages. Proteomic analyses revealed that grape flowers and berries display unique profiles of proteins involved in fatty acid biosynthesis, triacylglycerol assembly, fatty acid β-oxidation, and lipid signaling. These findings show, for the first time, that fatty acid metabolism also plays an important role in the development of non-oil-rich tissues, opening new perspectives about lipid function and its relation to berry quality.

## 1. Introduction

The grapevine (*Vitis vinifera* L.) is one of the most economically important fruit crops cultivated globally. There are more than 10,000 productive cultivars in the world, including table grapes and wine grapes [1]. Grape berries are consumed as fresh fruit and provide the raw material to produce wine, juices, and raisins [2]. The transformation of grape berries from hard, green structures to soft, mature, and flavorful fruits is a critical stage in their development [3]. Various biochemical and physiological changes occur during this process, including alterations in carbohydrates, organic acids, cell wall composition, and the less studied fatty acid and lipid metabolisms [4,5]. The ‘Thompson Seedless’ cultivar presents a reduction in seed content, a green-colored skin, and consumers’ desirable organoleptic traits, such as a high firmness and a balanced sweetness/acidity ratio [6]. Fruit quality in table grapes is mainly associated with berry firmness, and during grape development and ripening, cell wall components undergo modifications that significantly influence fruit firmness [7,8]. However, fruit firmness is not only given by the physical characteristics of the cell wall but also by the turgor of the berries, which is built by the interaction of water contained in cell spaces surrounded by membranes that exert force on the cell wall [9]. Therefore, the characteristics of the membranes and their integrity have a significant impact on fruit quality. In addition, grapes are a rich source of bioactive compounds, and recent advances in proteomic and metabolite profiling have provided valuable insights into the composition and health benefits of grapes and grape products, such as polyphenols, resveratrol, and anthocyanins [2,10]. Recent advances in proteomic and metabolite profiling have contributed to the understanding of the complex biology of grapevines and to improve grape production, wine quality, and vineyard sustainability [11,12]. However, only a few efforts have been made to integrate proteomic and metabolomic datasets, which have provided valuable information regarding the description of primary and secondary metabolisms of grape berries during development and ripening [5,11], the metabolic traits of anthocyanin accumulation in berry exocarp [13], and the effect of high temperatures on grape berry metabolism [14].

Understanding the underlying mechanisms of lipid accumulation and fatty acid transformations is essential for enhancing fruit quality and overall crop productivity. The metabolism of lipophilic compounds, which involve fatty acids that constitute the fundamental basis of membranes in plant tissues [15], usually undergo modifications associated with fruit quality [16,17,18,19]. In the literature, few references have focused on studying the metabolism of lipid compounds in table grapes during the early stages of development, concentrating on the period from veraison to harvest. In the literature, there is a special focus on varieties associated with wine production. It has been reported that in the grape cv. ‘Pedro Ximénez’, the overall content of saturated fatty acids increases with berry ripening, while the proportion of unsaturated acids decreases, with linoleic acid content being the most affected [20]. The catabolism of fatty acids mainly involves enzymes of the phospholipase, lipoxygenase, and peroxidase types [16,19,21], which induce changes in the proportion of these lipophilic compounds and the physicochemical characteristics of the fruit membranes. On the other hand, during grape ripening, the biosynthesis of fatty acids, especially saturated fatty acids such as octacosanoic, hexacosanoic, and triacontanoic acids, shows an increase associated with the synthesis of waxes and aroma-related compounds [22].

Fatty acids are fundamental components of lipids, serving as building blocks for various lipid classes such as triacylglycerols (TAGs), phospholipids, and sterols [23]. De novo fatty acid biosynthesis occurs primarily within the plastids of grape berry cells [24]. Acetyl-CoA carboxylase (ACC) and the fatty acid synthase (FAS) complex are key enzymes involved in this process. The malonyl-CoA produced by ACC condenses with acetyl-CoA, elongating the fatty acid chain [24]. During early grape berry development, the expression levels of genes encoding ACC and FAS increase significantly, supporting the synthesis of fatty acids and the production of essential lipids for cellular functions [25,26]. The production of long-chain fatty acids is crucial for forming storage lipids like TAGs, which serve as energy reservoirs during grape berry ripening [27]. Fatty acid β-oxidation is a fundamental metabolic process that involves the breakdown of fatty acids to generate energy through the production of acetyl-CoA units, which can be further used in the Krebs cycle to produce cellular energy. In plants, this process primarily occurs in peroxisomes/glyoxysomes, specialized organelles responsible for various metabolic reactions, including the breakdown of fatty acids [28].

Lipids are precursors of signaling molecules, such as jasmonates, that regulate stress responses and defense mechanisms during grape berry ripening [29,30]. Plant lipid signaling is pivotal in coordinating several physiological processes essential for growth, development, and responses to environmental cues [31]. Lipids, as essential components of cellular membranes, have long been recognized for their structural roles, but their involvement in signaling pathways has gained increasing attention [32].

As a continuation of previous work focused on grape berry central carbon metabolism [5], the aim of this study is to understand the molecular mechanisms involved in fatty acid metabolism in table grapes, which is closely related to central carbon metabolism. We guided our proteomic and low-polar metabolite profiling analyses on the lipid metabolism of ‘Thompson Seedless’ and elucidated the dynamics during flower and berry development, providing novel information on table grape fatty acids fluctuation.

## 2. Results and Discussion

### 2.1. Lipid Profiling of Grape Bunches during Flowering and Berry Development

**Figure 1:**
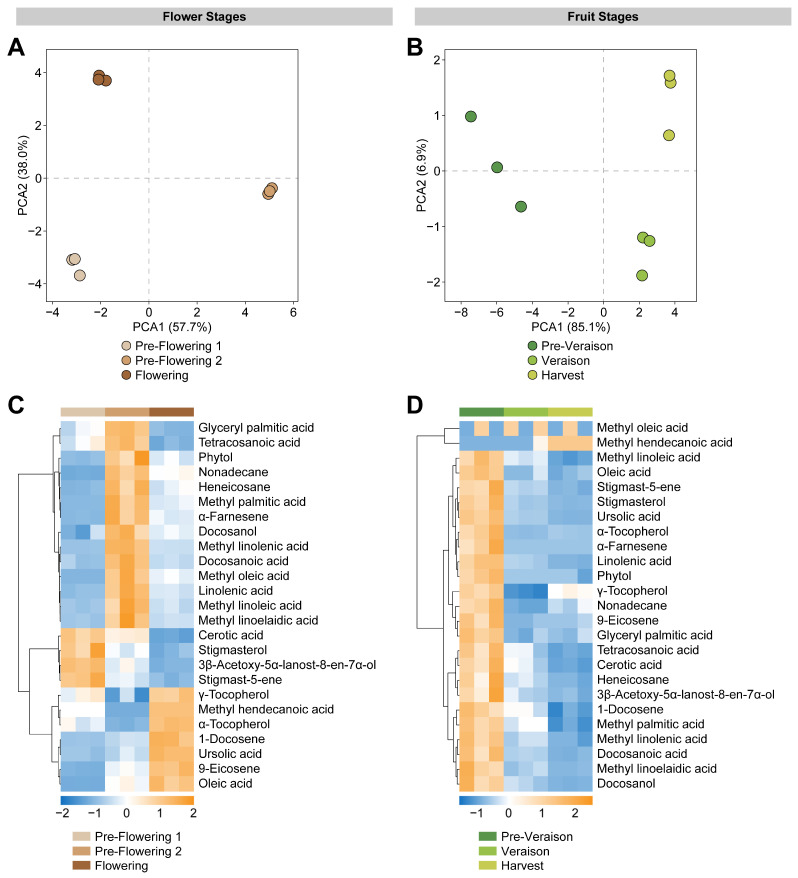
Multivariate analyses of lipid metabolites of grape bunches at flower and fruit stages. Principal component analysis (PCA) score plot for flower stages (**A**) and fruit stages (**B**). Heatmap representation based on 25 metabolites identified in grape bunches at pre-flowering 1, pre-flowering 2, and flowering stages (**C**), and at pre-veraison, veraison, and harvest stages (**D**). The columns represent biological replicates for each phenological stage. The similarity measure used to cluster the different features was based on Euclidean distance and Ward’s linkage from three biological replicates (*n* = 3).

Grape bunches previously used for polar metabolite profiling [5], were used for low-polar metabolite analysis by gas chromatography–mass spectrometry (GC-MS) and for a fatty acid methyl ester (FAME) profiling by gas chromatography–flame ionization detector (GC-FID) at pre-flowering 1, pre-flowering 2, flowering, pre-veraison, veraison, and harvest stages. A targeted low-polar metabolite profiling was performed from GC-MS analysis, and lipid compounds’ relative abundances were determined. Samples were assessed separately by comparing flower-related stages (pre-flowering 1, pre-flowering 2, and flowering) and fruit-related stages (pre-veraison, veraison, and harvest). Principal component analysis (PCA) score plots explained 95.7% of the variability with two components for flower stages (Figure 1A) and 92.0% for fruit stages (Figure 1B). PCA loading plots displayed the contribution of each metabolite to the variability at flower and fruit stages (Figure S1). In addition to these observations, visualization of the data using heatmaps containing the 25 compounds showed differential accumulation patterns for flowers and berries at different phenological stages. Four metabolites were accumulated in the pre-flowering 1 stage: cerotic acid; stigmasterol; 3β-acetoxy-5α-lanost-8-en-7α-ol; and stigmast-5-ene (Figure 1C). Interestingly, at pre-flowering 2, most of the compounds found elevated (14/25) were mainly fatty acids, such as glyceryl palmitate, tetracosanoate, methyl linolenate, docosanoate, methyl oleate, linolenate, methyl linoleate, and methyl linoelaidic acid (Figure 1C). At this stage prior to bloom, a peak in the accumulation of lipid-derived volatile compounds related to the bouquet of the grapevine flowers of the cv. ‘Cabernet Sauvignon’ and ‘Chardonnay’ have been described, including α-farnesene, nonadecane, and heneicosane [33,34]. At flowering, seven metabolites were found to be accumulated, including α-tocopherol, γ-tocopherol, methyl hendecanoic acid, ursolic acid, oleate, 1-docosene, and 9-eicosene (Figure 1C). Volatiles such as 1-docosene and 9-eicosene have been found in the essential oils of *Tamarix dioica* and *Rosa damascena* flowers, providing aroma traits when full bloom is achieved [35,36]. Tocopherols, lipid compounds known for their antioxidant activity and neurological protection [37], have been detected in grapevine flower tissues from the cv. ‘Alphonse Lavallee’; however, higher concentrations of α-tocopherol and γ-tocopherol were found in leaves and seeds [38]. 

For fruit stages, most of the low-polar metabolites accumulated in the early stage of berry development pre-veraison (Figure 1D). The accumulation pattern of volatile compounds has been described as highly dynamic during berry development and ripening [39]. We found that the volatile metabolite abundance abruptly diminished after pre-veraison. This trend has been previously described in the cv. ‘Viognier’, as the berries ripen and accumulate soluble solids, the volatile compound pool displayed a reduction in its amount [40]. α-tocopherol (and γ-) seems to have a cultivar-dependent accumulation, showing a decrease through ripening in our study (Figure 1D) and a decrease in the skin-colored berries of the cv. ‘Albert Lavallée’ [41], while on for the skin-colored cv. ‘Vinhao’, α-tocopherol exhibited higher levels in mature grape berries [25]. γ-tocopherol exhibited a decrease from pre-veraison to veraison and then an increase until ripening (Figure 1D). In addition, γ-tocopherol showed an increase from veraison to ripening in grape berries cv. ‘Albert Lavallée’ [41], and cv. ‘Vinhao’ [25]. Interestingly, at the ripening stage, we found that the relative amount of γ-tocopherol was slightly higher than α-tocopherol (Figure 1D), displaying an opposite accumulation, as described for several table grape and wine grape cultivars [42]. Stigmasterol abundance decreased during grape berry ripening, as previously reported for cv. ‘Vinhao’ [25]. Furthermore, grapevine genotype is not the only component influencing lipid profile. The role of environmental factors, such as warmer temperatures, modulate fatty acid and sterol abundance in grape chloroplasts, adding complexity to the study of lipid metabolism [43,44,45].

**Figure 2:**
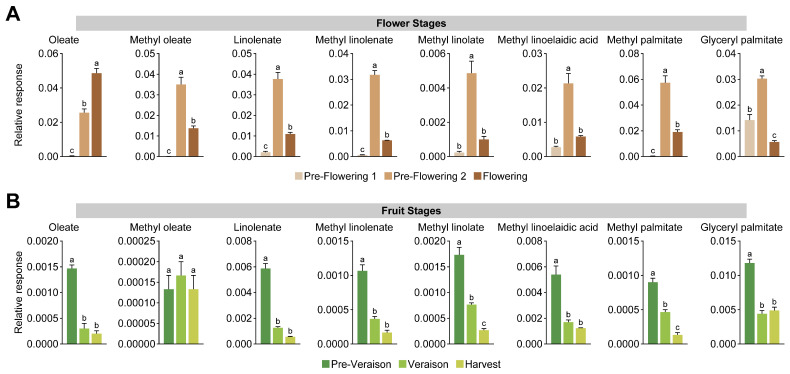
Univariate analysis of the relative abundance of the free fatty acids identified by GC-MS in grape bunches at flower stages (**A**) and fruit stages (**B**). Error bars represent SEM (*n* = 3). Data were analyzed by one-way ANOVA (different letters means *p* < 0.05).

**Figure 3:**
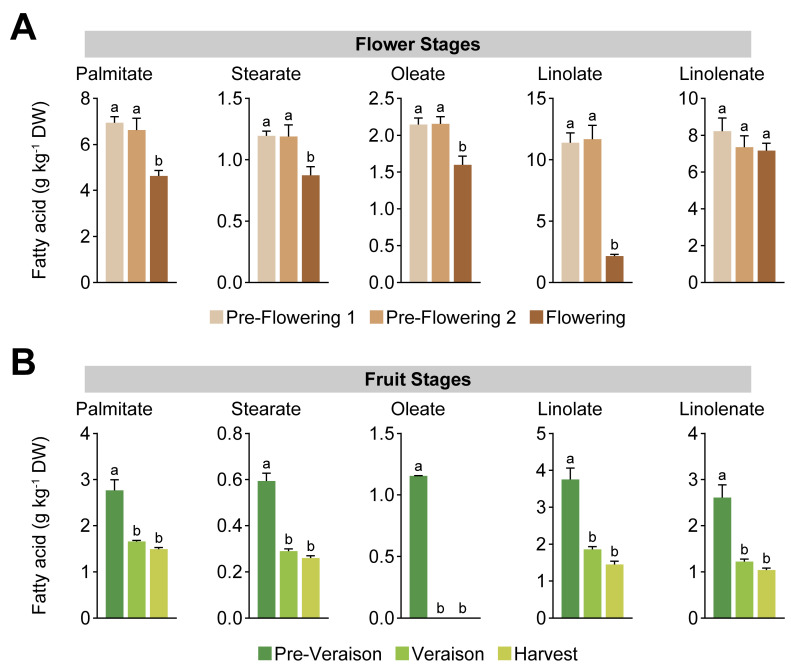
Univariate analysis of the quantified fatty acids by GC-FID in grape bunches at flower stages (**A**) and at fruit stages (**B**). Error bars represent SEM (*n* = 3). Data were analyzed by one-way ANOVA (different letters means *p* < 0.05).

In addition, a univariate analysis of the main free fatty acids detected by GC-MS and lipid-derived fatty acids by GC-FID was carried out. GC-MS analysis showed that oleate was highly accumulated during flowering (Figure 2A). On the other hand, methyl oleate, linolenate, methyl linolenate, methyl linolate, methyl linoelaidic acid, methyl palmitate, and glyceryl palmitate were accumulated in the pre-flowering 2 stage (Figure 2A). Most of these fatty acids displayed an abundance pattern in which there was a peak at the pre-flowering 2 stage, decreasing at flowering but remaining higher than at the pre-flowering 1 stage. For GC-FID analysis, lipid-derived fatty acids showed a similar pattern through flower development. However, there was an increased accumulation at pre-flowering 1\ and 2 stages, diminishing the quantity of fatty acids at flowering (Figure 3A). Prior to anthesis, cell division is particularly high, requiring increased levels of de novo-synthetized fatty acids for cell membrane assembly of the recently formed cells within floral tissues [46,47]. This could explain the drop in fatty acid content at flowering, in which, prior to this stage, the fatty acids were incorporated into cell membranes. In contrast, the free fatty acids could have been metabolized into the formation of volatile compounds required in floral scent [33].

For fruit stages, at pre-veraison, we observed higher concentrations of oleate, linolenate, methyl linolenate, methyl linolate, methyl linoelaidic acid, methyl palmitate, and glyceryl palmitate via GC-MS (Figure 2B). The fatty acid pattern accumulation indicates a decrease in these metabolites at veraison, remaining lower through the ripening process (Figure 2B). For GC-FID analysis, lipid-derived fatty acids showed a similar pattern through fruit ripening (Figure 3B), associated with the high growth rate of the berries at early stages of development, mainly characterized by cell division [3,48,49]. Through ripening, we observed lower levels of fatty acids, concomitant with a reduction in cell division rates, and the berry growth after veraison is determined by cell expansion, mobilizing higher amounts of water within the cells, explaining the decrease in the fatty acids required by the metabolic processes [26,50]. Previous studies between veraison and harvest using three grape cultivars indicated that lipophilic compounds, including fatty acids, showed a decreased concentration as the berry matures [22]. In addition, the integrity of cell membranes in grape berries of the cv. ‘Autumn Royal’ has been described to be related to some postharvest disorders, showing that the deterioration of table grapes due to dehydration and susceptibility to fungal infections depends on the composition of the membranes [51]. It has been reported that progressive disorganization of cell organelle membranes, associated with elevated rates of hydrogen peroxide generation, lipid peroxidation, and an imbalance in the saturated/unsaturated ratio of polar lipids, mainly due to a decrease in the degree of unsaturation of fatty acids, correlates with lower berry quality during postharvest storage [51]. Our data suggest that flowers and berries contain unique signatures in low-polar metabolite content, pointing to a differential accumulation during grape tissue development.

### 2.2. Proteomic Analysis of Grape Bunches during Flowering and Berry Development

**Figure 4:**
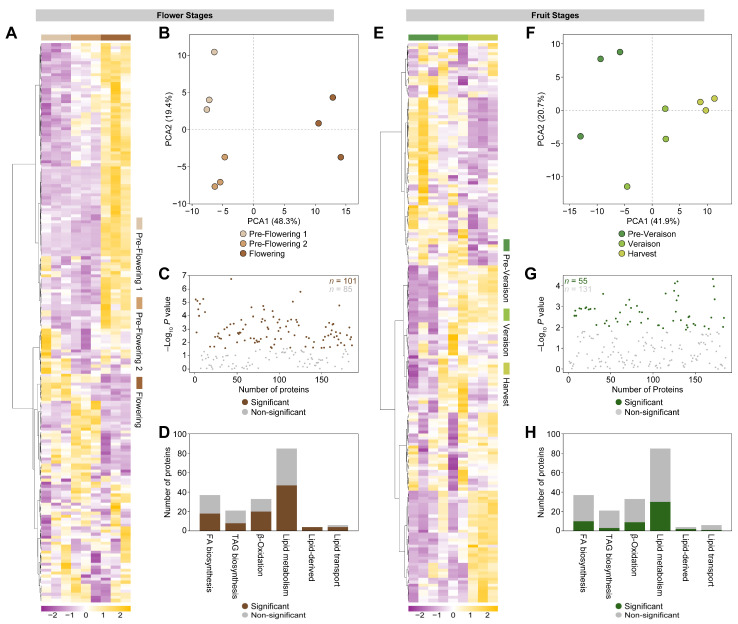
Multivariate analyses of grapevine proteins at pre-flowering 1, pre-flowering 2, flowering, pre-veraison, veraison, and harvest stages. Heatmap representation of the 186 identified proteins in grape bunches at flower stages (**A**) and fruit stages (**E**). The columns represent biological replicates for each phenological stage. The similarity measure used to cluster the different features was based on Euclidean distance and Ward’s linkage from three biological replicates (*n* = 3). PCA score plots for flower stages (**B**) and fruit stages (**F**). Significantly expressed proteins at flower stages (**C**; in brown) and fruit stages (**G**; in green) based on one-way ANOVA analysis (*p* < 0.05). Distribution of identified proteins and classification of lipid-related metabolism for flower stages (**D**) and fruit stages (**H**).

A proteomic approach was performed by liquid chromatography with tandem mass spectrometry (LC-MS/MS) to better understand the dynamics of lipid metabolism during grapevine flowering and fruit development. The proteomic dataset obtained was previously used for pathway analysis and reconstruction of grapevine central carbon metabolism [5]. For this work, we carried out data curation and focused on 186 lipid-related proteins that showed detectable abundance at the six phenological stages. Data visualization by heatmaps containing the complete list of lipid-related proteins showed a differential accumulation pattern for flowers and berries at different phenological stages (Figure 4A,E). It was possible to detect a large subset of proteins accumulated at the flowering stage. In contrast, the subgroups contained fewer proteins for pre-flowering 1 and pre-flowering 2 stages (Figure 4A). This differential pattern of protein accumulation was supported by a PCA analysis, where the score plot explained 67.7% of the variability with two components for flower stages (Figure 4B). A further analysis of variance (ANOVA) indicated that 101/186 (54.3%) of the proteins had a significantly differential expression in grapevine at floral stages (Figure 4C). Also, the identified proteins were classified into six groups: fatty acid (FA) biosynthesis; triacylglycerol (TGA) biosynthesis; FA β-oxidation; lipid metabolism; lipid-derived metabolism; and lipid transport. Figure 4D displays the distribution of these protein groups, indicating that enriched clusters correspond to lipid metabolism (mostly phospholipases), fatty acid biosynthesis, and FA β-oxidation.

For fruit stages, it was observed that a significant group of proteins were accumulated at the harvest stage, and an elevated number of these proteins were also increased at the veraison stage (Figure 4E). In addition, fewer proteins showed an increased abundance at the pre-veraison stage. The protein accumulation pattern suggested a similar dynamic of lipid-related proteins between the veraison and harvest stages, which was supported by PCA, observing that samples from these two phenological stages were similar (Figure 4F). The one-way ANOVA test indicated that 55/186 (29.6%) of the proteins had a significantly differential abundance in the grapevine at fruit stages (Figure 4G). Figure 4H complemented this finding, displaying a lower number of significant proteins by its classification. A previously described transcriptome during ripening of grape berries cv. ‘Thompson Seedless’ indicated that fatty acids and lipid metabolism are part of the most complex groups of genes showing several abundance patterns through the ripening process, including shifts of accumulation, depletion, or oscillations through berry ripening [26]. Proteins associated with lipid metabolism exhibited a higher abundance after veraison in grape berries cv. ‘Early Campbell’ [11]. On the other hand, in grape berries cv. ‘Cabernet Sauvignon’, identified genes related to lipid metabolism and showed a decreasing expression pattern across berry development [52].

### 2.3. Identification of Proteins Involved in Fatty Acid and TAG Assembly during Flowering and Berry Development

**Figure 5:**
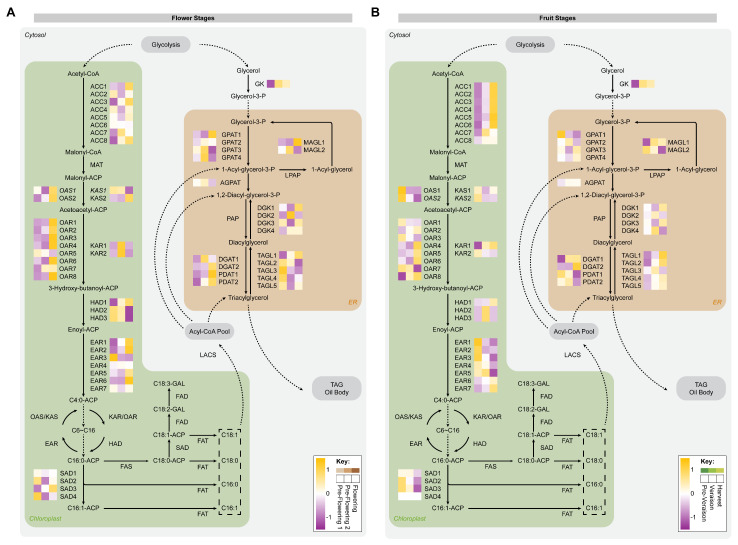
Fatty acid biosynthesis and TAG assembly pathway reconstruction. The map shows proteins (uppercase) and metabolites (lowercase) involved in the metabolic pathway at flower stages (**A**) and at fruit stages (**B**). Relative abundance of proteins was averaged over three biological replicates (*n* = 3). ACC, acetyl-CoA carboxylase; MAT, malonyl-CoA ACP transacylase; OAS, 3-oxoacyl-ACP synthase; KAS, ketoacyl-ACP synthase; OAR, 3-oxoacyl-ACP reductase; KAR, ketoacyl-ACP reductase; HAD, hydroxyacyl-ACP dehydrase; EAR, enoyl-ACP reductase; SAD, stearoyl-ACP desaturase; FAS, fatty acid synthase; *FAT*, acyl-ACP thioesterase; FAD, fatty acid desaturase; LACS, long-chain acyl-CoA synthetase; GK, glycerol kinase; GPAT, glycerol-3-phosphate acyltransferase; AGPAT, acyl-glycerol-3-phosphate acyltransferase; PAP, phosphatidic acid phosphatase; DGAT, diacylglycerol acyltransferase; PDAT, phospholipid:diacylglycerol acyltransferase; MAGL, monoacylglycerol lipase; LPAP, phosphatidate phosphatase; DGK, diacylglycerol kinase; TAGL, triacylglycerol lipase.

Fatty acid biosynthesis requires several enzymes and occurs within the chloroplast of plant cells [53]. A total of 36 proteins were identified in FA synthesis, and they were used for the reconstruction of the FA biosynthesis pathway. At flower stages, 47.2% (17/36) of the enzymes were significantly different. Most of these proteins (12/17) were found upregulated at the flowering stage, including acetyl-CoA carboxylases (ACC3 and ACC8), 3-oxoacyl-ACP reductases (OAR1, OAR3, OAR4, OAR6, and OAR8), 3-hydroxyacyl-ACP dehydratase (HAD1), enoyl-ACP reductases (EAR1, EAR2, and EAR6), and stearoyl-ACP 9-desaturase (SAD4; Figure 5A). At the pre-flowering 1 stage, EAR3, HAD2, and HAD3 were found to be more abundant, while at the pre-flowering 2 stage, the ketoacyl-ACP reductases (KAR1 and KAR2) displayed a protein accumulation (Figure 5A). During grapevine flower development, there is a lack of information related to proteomic and lipidomic analyses. At the grapevine flowering stage for cv. ‘Thompson Seedless’, a higher accumulation of the pyruvate dehydrogenase complex has been described compared to the pre-flowering 2 stage, suggesting an elevated production of acetyl-CoA that could be used as a precursor of malonyl-CoA, concomitant with the observed increase in the expression of ACC enzymes [5].

At fruit stages, 27.8% (10/36) of the enzymes were significantly different, and half of these proteins (5/10) were found to be more abundant at the pre-veraison stage, including EAR1, EAR3, OAR7, OAS1, and OAS2. At veraison, only the enzyme EAR3 was observed to be more abundant. For harvest stage, ACC5, ACC6, KAR1, and OAR8 were found to have accumulated (Figure 5B). Overall, our data suggest that fatty acid synthesis decreases during the ripening of grape berries, concomitant with the information obtained by GC-MS and GC-FID, where the accumulation of fatty acids also decreases. This behavior has been described in the cv. ‘Cabernet Sauvignon’ showing that the expression of genes involved in fatty acid synthesis decreases throughout berry ripening [54]. This study shows that the expression of some *KAS*, *KAR*, *HAD*, and *EAR* genes continuously decreases until grape berries reach maturity. Interestingly, we found proteins that shared this expression/abundance behavior and other proteins that exhibited an accumulation pattern throughout the ripening of grape berries. This behavior could be explained by the fact that fatty acids and their derivatives are involved in the signaling of metabolic pathways related to the ripening process of grape berries [54,55].

Related to TAG biosynthesis, little is known about this metabolic process in grapevine flower development. During flowering, some of the fatty acids pool are used for the synthesis of volatile compounds that confer the fragrance of the flowers [33,56]. However, it has been described that most of the TAGs are stored as an energy source [57]. A total of 21 proteins were identified in TAG synthesis and were used for the reconstruction of the TAG biosynthesis pathway. At flower stages, 38.1% (8/21) of the enzymes were significantly different and similarly distributed between pre-flowering 2 and flowering stages. Glycerol kinase (GK), glycerol-3-phosphate acyltransferase (GPAT3), and diacylglycerol kinase (DGK2) were found upregulated at the pre-flowering 2 stage, while GPAT1, phospholipid:diacylglycerol acyltransferases (PDAT1 and PDAT2), and monoacylglycerol lipase (MAGL1) showed an increased abundance at the flowering stage (Figure 5A). Interestingly, during the flower development of *Camellia reticulata*, transcriptomic analysis showed the same accumulation pattern observed in our data for DGAT1, DGAT2, PDAT1, and PDAT2 [58].

At fruit stages, 14.3% (3/21) of the enzymes were significantly different, and GPAT1 was found upregulated at the pre-veraison stage, while GK and DGAT1 were observed accumulated at veraison (Figure 5B). In agreement with our findings, during the ripening of grape berries of the cv. ‘Vinhao’, it has been observed that *GPAT-* and *DGAT*-transcript expression decreased across ripening [25]. In addition, the TAG synthesis pathway displayed an elevated transcriptional activity during the ripening of grape berries cv. ‘Thompson Seedless’, concomitant with our findings in which we observed an increase in GPATs, 1-acyl-glycerol-3-phosphate acyltransferase (AGPAT), and DGATs protein accumulation [26]. In addition, in grapes cv. ‘Airen’ and ‘Cencibel’, the TAG content oscillates during late stages of ripening, displaying peaks of TAG accumulation close to the end of ripening, and showed a differential abundance between both cultivars, being higher in the cv. ‘Cencibel’ [59]. This metabolic overall upregulation of TAG across berry maturation could explain the decrease in free fatty acid content, suggesting a coordinated regulation of lipid metabolism during grape berry ripening.

### 2.4. Fatty Acid β-Oxidation Dynamics during Flowering and Berry Development

**Figure 6:**
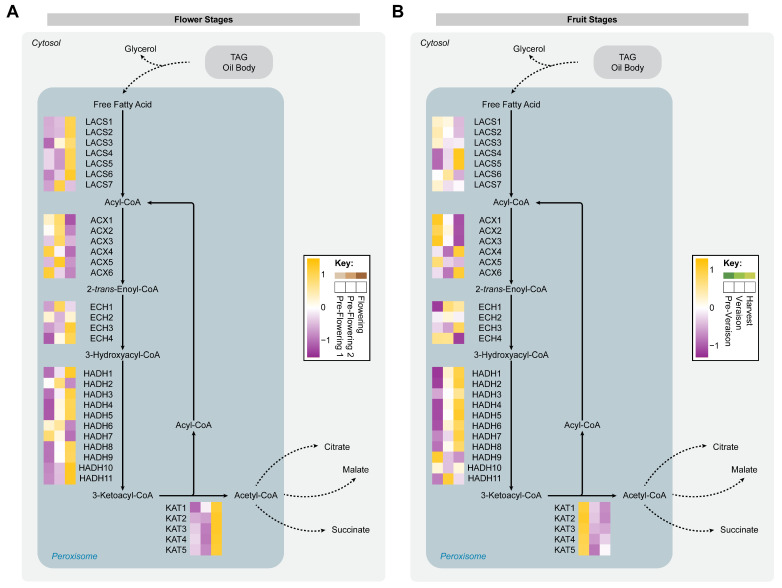
Fatty acid β-oxidation pathway reconstruction. The map shows proteins (uppercase) and metabolites (lowercase) involved in the metabolic pathway at flower stages (**A**) and at fruit stages (**B**). Relative abundance of proteins was averaged over three biological replicates (*n* = 3). LACS, long-chain acyl-CoA synthetase; ACX, acyl-CoA oxidase; ECH, enoyl-CoA hydratase; HADH, 3-hydroxyacyl-CoA dehydrogenase; KAT, ketoacyl-CoA thiolase.

Free fatty acids derived from TAGs are mainly metabolized within the peroxisome/glyoxysome via β-oxidation [28]. A total of 33 proteins were identified related to β-oxidation and they were used for the reconstruction of this metabolic pathway. At flower stages, 60.6% (20/33) of the proteins were significantly different and most of these enzymes (16/20) were found to be more abundant at the flowering stage, including long-chain acyl-CoA synthetase (LACS6), enoyl-CoA hydratases (ECH3 and ECH4), 3-hydroxyacyl-CoA dehydrogenases (HADH1, HADH3, HADH4, HADH5, HADH8, HADH9, HADH10, and HADH11), and 3-ketocayl-CoA thiolases (KAT1, KAT2, KAT3, KAT4, and KAT5); Figure 6A. At the pre-flowering 1 stage, acyl-CoA oxidases (ACX4 and ACX6) were found to be more abundant, while at the pre-flowering 2 stage, the ACX1 and ACX5 displayed a protein accumulation (Figure 6A). A metabolic pathway closely related to fatty acid β-oxidation is the glyoxylate cycle pathway, where acetyl units are converted into four-carbon acids to produce sugars by gluconeogenesis [60]. The acetyl-CoA derived from fatty acid β-oxidation could be used for citrate and malate production through citrate synthase and malate synthase enzymes in the glyoxylate cycle [61]. Interestingly, during flowering in cv. ‘Thompson Seedless’, a higher accumulation of citrate and malate compared to the pre-flowering 2 stage has recently been reported, suggesting a coordinated utilization of fatty acid-derived acetyl-CoA for energy production in that stage of flower development with elevated cell division and metabolism [5,49].

At fruit stages, 27.3% (9/33) of the enzymes showed a significantly different accumulation, and most of these proteins (6/9) were found to be more abundant at the harvest stage, including LACS4, LACS6, HADH1, HADH2, HADH4, and HADH5. For the pre-veraison stage, ACX1 and ACX3 were found to have accumulated. At veraison, only the enzyme ECH1 was observed to be more abundant (Figure 6B). Our findings suggest that fatty acid β-oxidation increases during ripening of grape berries, supported by the information obtained by GC-MS and GC-FID, where the accumulation of fatty acids diminishes. Degradation of thylakoid membranes is an important manifestation of cellular senescence during the late stages of ripening, and it is characterized by an increment in TAG disassembly and fatty acid oxidation [62,63]. In addition, a transcriptome analysis suggested that lipid catabolism increases during ripening of grape berries cv. ‘Thompson Seedless’ by a continuous increase in the expression of genes associated with fatty acid β-oxidation [26]. This analysis showed that enoyl-CoA hydratase transcripts were particularly upregulated through the ripening process, and our data showed a similar accumulation pattern for the ECH3 enzyme. On the other hand, the malate and citrate abundance showed a peak amount at the veraison stage, suggesting an alternative use of the acetyl-CoA produced from fatty acid β-oxidation at the harvest stage [5].

### 2.5. Lipid Metabolism and Signaling during Flowering and Berry Development

**Figure 7:**
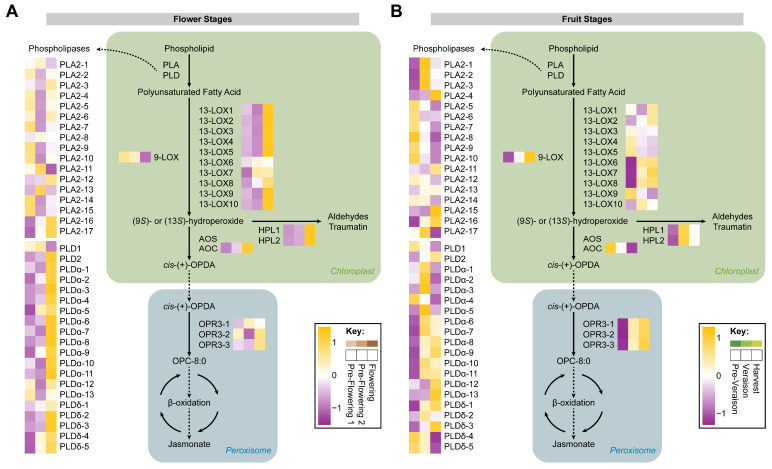
Lipoxygenase pathway reconstruction. The map shows proteins (uppercase) and metabolites (lowercase) involved in the metabolic pathway at flower stages (**A**) and at fruit stages (**B**). Relative abundance of proteins was averaged over three biological replicates (*n* = 3). *PLA*, phospholipase A; *PLD*, phospholipase D; *9-LOX*, linoleate 9*S*-lipoxygenases; *13-LOX*, linoleate 13*S*-lipoxygenases; *AOS*, allene oxide synthase; *AOC*, allene oxide cyclase; *HPL*, hydroperoxide lyase; *OPR3*, 12-oxophytodienoate reductase.

Plant lipid signaling represents a dynamic system that orchestrates a wide range of cellular processes. From structural components of membranes to secondary messengers and regulators of stress responses, lipids play integral roles in plant growth, development, and adaptation to changing environments [30,64]. Lipid peroxidation is carried out through the lipoxygenase pathway and has been described as the main polyunsaturated fatty acid processing pathway [65]. From this fatty acid dioxygenation, several enzymes participate in the formation of numerous lipid-derived signaling molecules, with jasmonate, traumatin, and hexenal being the most studied oxylipins [66]. To understand lipid signaling in grapevines, a partial reconstruction of the lipoxygenase pathway was carried out. A total of 54 proteins were identified for this pathway and at flower stages, 53.7% (29/54) of the enzymes were significantly different and most of these proteins (28/29) were found upregulated at flowering stage, including 19 phospholipases (PLA2-16, PLA2-17, PLD2, PLDα-1, PLDα-2, PLD3α-3, PLDα-4, PLDα-5, PLDα-6, PLDα-7, PLDα-8, PLDα-9, PLDα-10, PLDα-11, PLDδ-2, PLDδ-3, PLDδ-4, and PLDδ-5), linoleate 13*S*-lipoxygenases (13-LOX1, 13-LOX2, 13-LOX3, 13-LOX4, 13-LOX5, 13-LOX9, and 13-LOX10), allene oxide cyclase (AOC), and hydroperoxide lyases (HPL1 and HPL2) (Figure 7A). During flowering, it has been described that jasmonate is required for the normal transition of flower development and has a role in controlling flowering time, concomitant with the observed upregulation of proteins involved in jasmonate biosynthesis, such as 13-LOXs, AOC, and β-oxidation enzymes [67,68]. Additionally, the (*Z*)-3-hexenal produced by hydroperoxide lyase activity has been reported to be highly concentrated at full bloom in grapevines cv. ‘Chardonnay’, displaying a correlation with the HPL protein accumulation detected during flowering [34,69]. Phospholipases also play critical roles in flower development. A previous work revealed that PLA1 is involved in flower formation and catalyzes the first step in jasmonate production [70]. Moreover, it has been described that PLA2 accumulates gradually during flower development, concomitant with our findings for *PLA2-16* and *PLA2-17* expression [71,72]. In addition, a recent study demonstrated that PLDα is required for proper flower development and pollination, accumulating stigmas during flowering, supporting the data obtained [73]. 

At fruit stages, 33.3% (18/54) of the proteins exhibited a significantly different accumulation, and most of these proteins (10/18) were found to be more abundant at the harvest stage, including lipoxygenases (13-LOX6, 13-LOX7, 13-LOX8, and 9-LOX), 12-oxophytodienoate reductases (OPR3-1, OPR3-2, and OPR3-3), and phospholipases (PLA2-16, PLA2-16, PLDδ-1). For veraison stage, PLA2-1, PLA2-2, PLA2-3, PLA2-17, and PLDα-5 were found to have accumulated. At pre-veraison, the enzymes AOC, PLA2-8, and PLDδ-4 were observed to be more abundant (Figure 7B). Jasmonate has been reported to show a peak of abundance a couple of weeks before veraison and then gradually diminishes until harvest in table grapes cv. ‘Fujiminori’ [29]. However, we speculate that jasmonate could progressively accumulate until mature stages in grape berries cv. ‘Thompson Seedless’ based on 13-LOX and OPR3 protein abundance patterns. Additionally, linoleate 13S-lipoxygenase increases during ripening of grape berries cv. ‘Blanc du Bois’ [74] and cv. ‘Thompson Seedless’ [26]. Furthermore, a hydroperoxide lyase gene characterized in grape berries cv. ‘Cabernet Sauvignon’ showed an expression pattern similar to our data with a peak of *HPL* accumulation close to veraison [75]. Hydroperoxide lyase was found to be slightly upregulated from the green to ripe stages, while *PLDα* exhibited an abundance reduction in grape berries cv. ‘Noble’ [76]. Moreover, lipoxygenase expression in grape berries cv. ‘Cabernet Sauvignon’ showed an increment in two LOX1 genes, while for another *LOX1*, two *LOX2*, and a *LOX* gene, a reduction in the expression was reported, pointing to a highly dynamic spatiotemporal lipoxygenase expression pattern, as observed in our findings [52,54].

## 3. Materials and Methods

### 3.1. Plant Material and Phenotypical Analyses

Grape (*Vitis vinifera* L.) inflorescences and bunches of the cv. ‘Thompson Seedless’ were collected and phenotypically characterized as previously described by Olmedo et al. [5] during the 2019–2020 growing season at a vineyard located in Lampa (33°20′49.3″ S, 70°53′11.8″ W), Metropolitan Region, Chile. Samples were harvested at six Eichhorn and Loren (E-L) growth stages [77]: pre-flowering 1 (E-L 12); pre-flowering 2 (E-L 15); flowering (E-L 19); pre-veraison (E-L 29); veraison (E-L 35); and harvest (E-L 38).

### 3.2. GC-MS Low-Polar Metabolite Analysis

Extraction and derivatization were performed according to Lytovchenko et al. [78] with modifications. Briefly, low-polar metabolites were extracted from 50 mg of lyophilized flower or berry tissues using 1.3 mL of HPLC-grade methanol. Samples were incubated in a thermoregulated shaker at 120 rpm for 15 min at 70 °C. After cooling using icepacks, the samples were centrifuged at 17,000× *g* for 10 min at 4 °C. From the extract obtained, 0.75 mL were transferred to a centrifuge tube, and a second extraction was undertaken by adding 1 mL of HPLC-grade methanol to the centrifuged sample. The incubation and centrifugation steps were then repeated. One milliliter of this new extraction was added to point seven, five milliliters of the previously saved extraction. Then, 1 mL of HPLC grade water and 1 mL of the internal standard (0.52 g L^−1^ of methyl undecanoate in chloroform) were added, and the samples were vigorously shaken, followed by centrifugation at 4800× *g* for 10 min at 4 °C. The upper phase was discarded, and 2 mL of methanol:water (1:1) was added. Samples were vortexed and incubated at 4 °C overnight. After incubation, samples were centrifugated at 4800× *g* for 10 min at 4 °C. The upper polar phase was discarded, and the lower chloroform phase was evaporated using gaseous nitrogen. Then, 0.2 mL of *N*-trimethylsilyl-*N*-methyl trifluoroacetamide (MSTFA) was added to the samples, followed by incubation at 120 rpm for 30 min at 37 °C. Samples were collected and transferred to GC vials. The vials were capped and stored at −80 °C until further GC analysis.

The low-polar metabolites were determined by injecting 1 µL of the prepared samples into an Agilent 7890B gas chromatograph equipped with a 5977A single quadrupole mass spectrometer, an electron impact ionization source, a PAL3 autosampler, and a 30 m × 0.25 mm × 0.25 µm DB-5ms column (Agilent Technologies, Santa Clara, CA, USA). The injector and interface temperatures were 220 °C and 280 °C, respectively. The helium flow rate was 1 mL min^−1,^ and a 25:1 split ratio was used. The initial temperature of the oven was 120 °C for 1 min; then, it was increased to 300 °C (5 °C each 1 min) and maintained for 15 min. The ionization source and quadrupole temperatures were 230 °C and 150 °C, respectively. Mass spectra were obtained in a 50 to 600 m/z interval with a scan rate of 2.66 cycles per second. Metabolites were identified using MassHunter Quantitative Analysis software version B.07.01 (Agilent Technologies, Santa Clara, CA, USA) and the NIST Library14. Metabolites were reported based on their relative quantification. QC samples composed of equal amounts of all samples were run every 10 samples. To obtain a relative response of each compound, the peak area data were corrected using the peak area of phenyl β-D-glucopyranoside (as an internal standard), the sample fresh weight, and a quality control (QC) sample representative of all samples. Data were analyzed by using relative concentration tables in the MetaboAnalyst 5.0 software. The software indicated that a total of 0% of missing values were detected. The variables were mean-centered and weighted using standard deviations to assign an equal variance. The metabolites were reported as previously described [79] in Table S1.

### 3.3. GC-FID Fatty Acid Analysis

The extraction was performed using a previous method with some modifications [80]. Briefly, fatty acids were extracted from 50 mg of lyophilized flower or berry tissues using 0.07 mL of 10 N KOH in HPLC-grade water and 0.53 mL of HPLC-grade methanol. Samples were incubated in a water bath for 1.5 h at 58 °C with vigorous shaking every 30 min. After cooling to room temperature in a cold tap water bath, 0.058 mL of 24 N H_2_SO_4_ was added. The tubes were then mixed by inversion and incubated again in the water bath for 1.5 h at 55 °C with shaking every 30 min. After fatty acid methyl ester (FAME) synthesis, the tubes were cooled in a cold tap water bath. Then, 0.5 mL of hexane and 0.01 mL of internal standard (methyl undecanoate: 26,16 g L^−1^) were added in a proportion of 0.97 mL of hexane and 0.03 mL of the internal standard. The tubes were vortexed for 2 min and centrifuged for 10 min at 17,000× *g*. The hexane phase (upper phase) containing the FAME was collected and transferred to GC vials. The vials were capped and stored at −80 °C until further GC analysis.

The fatty acid composition of the FAME fraction was determined in a gas chromatograph (GC-2014 Shimadzu, Kyoto, Japan) equipped with a capillary column (RTX 2330, 90% biscyanopropyl/10% phenylcyanopropyl polysiloxane, 105 m × 0.25 mm ID, 0.20 μm film thickness; Restek, Bellefonte, PA, USA). The injector temperature was set to 220 °C; flame ionization detector (FID) at 280 °C. Helium was used as carrier gas with the flow at 1 mL min^−1^. The column heating ramp was initially set at 130 °C, held for 5 min, followed by an increase from 130 °C to 180 °C at 5 °C per min, held at 180 °C for 10 min, increased from 180 °C to 240 °C at 3 °C per min, and held at 240 °C for 13 min. For fatty acid identification and quantification, the retention times were compared to the external analytical standards. The results were expressed as g of fatty acid kg^−1^ dry weight (DW). 

### 3.4. Extraction and Digestion of Proteins

Protein extraction and digestion were performed as previously described with some modifications [81]. Samples were mixed using 200 mg of frozen flower or berry tissues in 0.5 mL of cold extraction buffer (100 mM Tris–HCl pH 8.5, 5 mM ethylenediaminetetraacetic acid (EDTA; Sigma-Aldrich, St. Louis, MO, USA), 100 mM potassium chloride (KCl), 1% (*w*/*v*) dithiothreitol (DTT; Sigma-Aldrich, St. Louis, MO, USA), 1 mM phenylmethylsulfonyl fluoride (PMSF; Sigma-Aldrich, St. Louis, MO, USA), and 30% (*w*/*v*) sucrose, and immediately vortexed. Then, 0.5 mL of tris-equilibrated phenol solution was added, and the samples were homogenized for 10 min at 4 °C and centrifuged at 10,000× *g* for 10 min at 4 °C. The phenolic phase was collected and re-extracted by adding 0.5 mL of extraction buffer and centrifuged again. The collected phenolic phase was incubated overnight at −20 °C to induce protein precipitation with the addition of 1 mL of 0.1 M ammonium acetate in methanol. After incubation, samples were centrifuged at 17,000× *g* for 30 min at 4 °C, and the supernatant was discarded. Then, the pellet was rinsed using 0.2% (*w*/*v*) DTT in cold acetone, incubated at −20 °C for 1 h, and centrifuged again. The pellet obtained was air-dried, resuspended in 0.1 mL of lysis buffer (8 M urea, 5 mM DTT, and 30 mM Tris), and dissolved by vigorous agitation. Protein quantification was carried out with a Bio-Rad Protein Assay (Bio-Rad Inc, Hercules, CA, USA), according to the manufacturer’s instructions, and using bovine serum albumin as the protein standard. Then, 0.02 mg of proteins were incubated for 15 min with DTT solution up to a final concentration of 20 mM. The samples were incubated for 30 min in the dark with iodoacetamide up to a final concentration of 50 mM and diluted using 0.15 M ammonium bicarbonate. The digestion of proteins was carried out by adding trypsin (0.2 mg mL^−1^) and incubated overnight at 37 °C. Peptides were acidified with trifluoroacetic acid (TFA; Sigma-Aldrich, St. Louis, MO, USA) solution (0.1% [*v*/*v*] final concentration) and purified using Pierce C18 spin columns (Thermo Scientific, Rockford, IL, USA). The solvent was evaporated by Speed-Vac evaporation (Eppendorf, Hamburg, Germany), and the pellet was dissolved in 0.1 M ammonium formate.

### 3.5. LC-MS/MS Gel Free Proteomic Analysis

Samples were separated from 0.5 μg of digested material using an Ultimate 3000 ultra-high performance liquid chromatography (UHPLC) system (Dionex, Thermo Scientific, Rockford, IL, United States) equipped with an Acclaim PepMap100 C18 pre-column (3 μm, 100 A, Thermo Scientific, Rockford, IL, United States) and a C18 PepMap RSLC column (2 μm, 50 μm–15 cm, Thermo Scientific, Rockford, IL, USA). The mobile phase was composed of solvent (A), 0.1% (*v*/*v*) formic acid (Sigma-Aldrich, St. Louis, MO, USA), in water and solvent (B), 0.08% (*v*/*v*) formic acid, in 80% (*v*/*v*) acetonitrile (Sigma-Aldrich, St. Louis, MO, USA), using a linear gradient (0.3 mL min^−1^) of 0–4% buffer B (80% (*v*/*v*) acetonitrile, 0.08% (*v*/*v*) formic acid) in 3 min, 4–10% B in 12 min, 10–35% in 20 min, 35–65% in 5 min, 65–95% in 1 min, 95% for 10 min, 95–5% in 1 min, and 5% in 10 min [82]. The mass spectra were acquired using a Q Exactive hybrid quadrupole-Orbitrap mass spectrometer (Thermo Fisher Scientific, Rockford, IL, USA) operated in positive ion mode with a nanospray voltage of 2.1 kV and a source temperature of 250 °C. A Proteo Mass LTQ/FT Hybrid ESI Pos. Velos ESI positive ion calibration mix (88323, Thermo Scientific, Rockford, IL, USA) was used as an external calibrant. The instrument was operated in the data-dependent acquisition (DDA) mode with a survey MS scan at a resolution of 70,000 (fw hm at *m*/*z* 200) for the mass range of *m*/*z* 400 to 1600 for precursor ions, followed by MS/MS scans of the top ten most intense peaks with 2+, 3+, 4+, and +5 charged ions above a threshold ion count of 16,000 at 17,500 resolution using a normalized collision energy of 25 eV with an isolation window of 3.0 *m*/*z* and dynamic exclusion of 10 s. Data were acquired with Xcalibur 3.1.66.10 software (Thermo Scientific, Rockford, IL, USA). All raw data were converted into mgf files by Proteome Discoverer 1.4 (Thermo Fisher Scientific, Rockford, IL, USA) for identification and processed using Mascot 2.2.06 (Matrix Science, London, United Kingdom) against a Vitis vinifera cv. ‘Cabernet Sauvignon’ clone 08 v1.1–Chromosome scale database [83]. The parameters used to search in Mascot corresponded to a parent tolerance of 10 ppm, fragment tolerance of 0.02 Da, variable modification oxidation of M, fixed modification with carbamidomethyl C, and up to one missed cleavage for trypsin. Results were imported into Scaffold 3.6.3, and protein identification retained those proteins containing at least one identified peptide with a confidence level of 95%, a resulting false discovery rate of 0.0%, and the identification of a total of 4166 proteins.

### 3.6. Statistical Analyses

Principal component analysis (PCA) was performed on the normalized dataset obtained by GC-MS, GC-FID, and LC-MS/MS using the MetaboAnalyst 5.0 software. A one-way ANOVA with a Fisher’s least significant difference (LSD) post-hoc test was used to compare the means of each metabolite or protein at different phenological stages. The ANOVA was performed using R software version 4.0.2 (Vienna, Austria), with significance set at *p* < 0.05, and was conducted using the ‘agricolae’ package. Experiments were carried out using three biological replicates (*n* = 3).

## 4. Conclusions

In this study, our results focused, for the first time, on the dynamics of fatty acid metabolism during flower and table grape berry development. We found that flowers and berries contain unique signatures in low-polar metabolite content, indicating a differential accumulation pattern during grape tissue development. Also, our data showed an overall increased fatty acid biosynthesis at the late stages of flower development and the early stages of berry growth, concomitant with the required lipids by the active cell division rates. In addition, an increased fatty acid catabolism by β-oxidation was found during berry ripening, suggesting an important role for fatty acid metabolism in energy production through berry development. Moreover, higher lipid signaling, closely related to the formation of jasmonate by fatty acid β-oxidation during the late stages of berry ripening, proposes a maturity process accompanied by senescence of the tissue. Finally, this work provides novel information about the role of lipid metabolism in non-oil-rich tissues during grapevine development and ripening, opening new perspectives on and future research outlines into the function of fatty acids in maintaining the homeostasis of energy production during the growth and development of grapevines and their relation to fruit quality.

## Data Availability

Not applicable.

## Supplementary Materials

The following supporting information can be downloaded at: https://www.mdpi.com/article/10.3390/ijms242015360/s1.
