# Supplementary material for: Proteomic and Low-Polar Metabolite Profiling Reveal Unique Dynamics in Fatty Acid Metabolism during Flower and Berry Development of Table Grapes
Source: Int J Mol Sci. 2023 Oct 19;24(20):15360. doi: 10.3390/ijms242015360 (PMC10607693; doi:10.3390/ijms242015360)
Supplement: Supplementary file 1 [file ijms-24-15360-s001.zip › Figure S1.pdf]

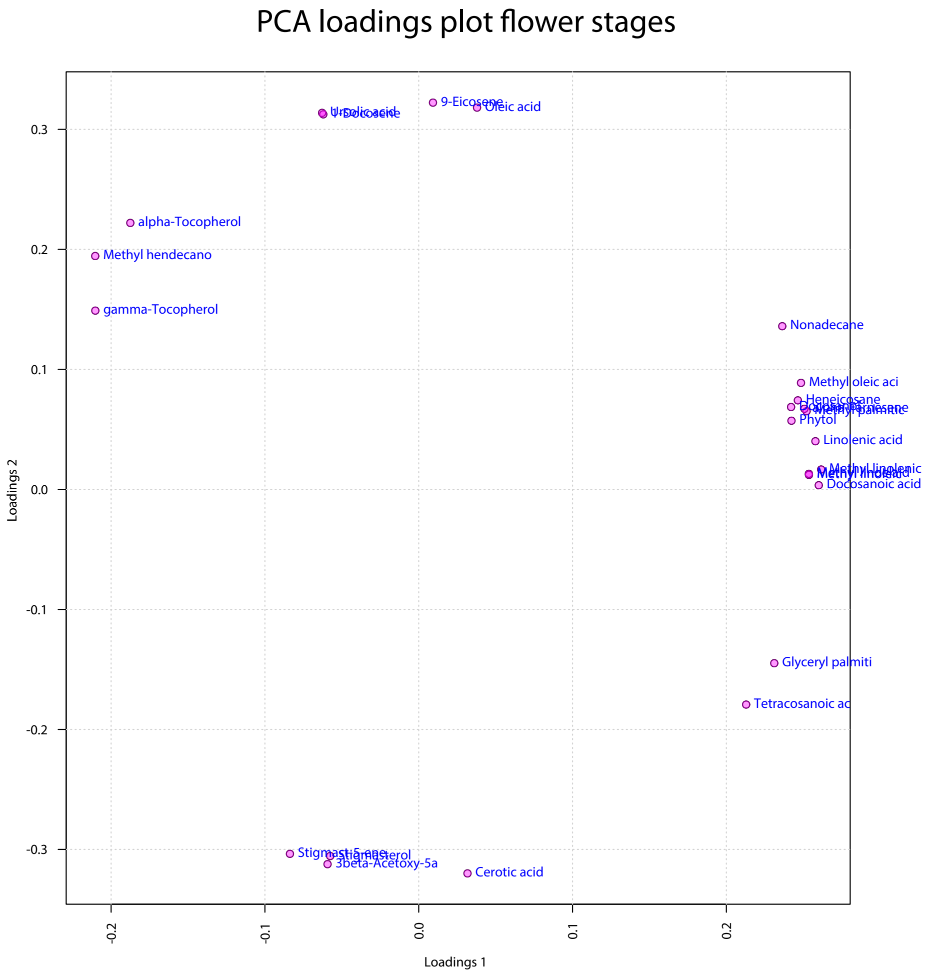

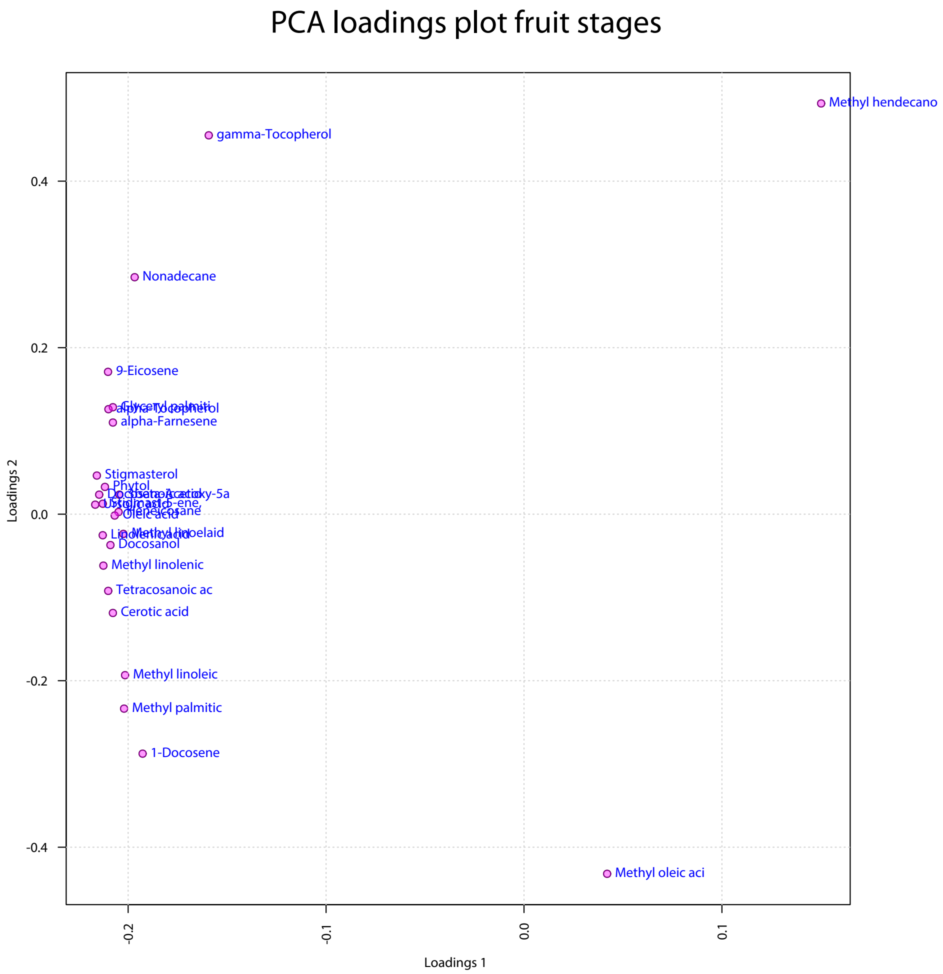

Figure S1: PCA loadings plots for low-polar metabolites at flower and fruit stages.
