# Supplementary material for: Proteomic and Low-Polar Metabolite Profiling Reveal Unique Dynamics in Fatty Acid Metabolism during Flower and Berry Development of Table Grapes
Source: Int J Mol Sci. 2023 Oct 19;24(20):15360. doi: 10.3390/ijms242015360 (PMC10607693; doi:10.3390/ijms242015360)
Supplement: Supplementary file 1 [file ijms-24-15360-s001.zip › Table S1.pdf]

Peak no.	RT	Metabolite name	Metabolite class	Molecular formula	ES(+) theor-m/z	ES(+) theor-m/z	ES(-) found m/z	ES(-) theor-m/z	m/z error (ppm)	MS/MS ES(+) fragments	MS/MS (+) CE (eV)	Reference ID	Identification level (A-D)
1	7.617	Undecanoic acid, methyl ester	Fatty acids	$C_{12}H_{24}O_2$						74<br>87<br>55		15607	
2	9.251	α-Farnesene	Terpenes	$C_{15}H_{24}$						93<br>69<br>55		5281516	
3	17.380	Nonadecane	Alkanes	$C_{19}H_{40}$						57<br>71<br>85		12401	
4	17.845	Hexadecanoic acid, methyl ester	Fatty acids	$C_{17}H_{34}O_2$						74<br>87<br>55		8181	
5	20.677	9-Eicosene	Alkanes	$C_{20}H_{40}$						55<br>97<br>83		5365037	
6	20.780	1-Docosene	Alkenes	$C_{22}H_{44}$						55<br>57<br>97		74138	
7	20.999	9,12-Octadecadienoic acid, methyl ester	Fatty acids	$C_{19}H_{34}O_2$						67<br>81<br>55		5284421	
8	21.102	9,12,15-Octadecatrienoic acid, methyl ester	Fatty acids	$C_{19}H_{32}O_2$						79<br>67<br>95		5367462	
9	21.177	Heneicosane	Alkanes	$C_{21}H_{44}$						57<br>74<br>85		12403	
10	21.238	trans-13-Octadecenoic acid, methyl ester	Fatty acids	$C_{19}H_{36}O_2$						55<br>69<br>74		5364432	
11	22.433	Phytol	Terpenes	$C_{23}H_{48}OSi$						143<br>73<br>75		5372684	
12	23.080	Linoelaidic acid, trimethylsilyl ester	Fatty acids	$C_{21}H_{40}O_2Si$						73<br>75<br>67		5366424	
13	23.184	α-Linolenic acid	Fatty acids	$C_{21}H_{38}O_2Si$						73<br>75<br>79		5366426	
14	23.314	Oleic Acid	Fatty acids	$C_{21}H_{42}O_2Si$						73<br>75<br>117		5366433	
15	28.664	Docosanol	Fatty acids	$C_{25}H_{54}OSi$						383		529830	

										75<br>73			
16	29.212	1-Monopalmitin	Fatty acids	$C_{25}H_{54}O_4Si_2$						371<br>73<br>147		552917	
17	29.997	Behenic acid	Fatty acids	$C_{25}H_{52}O_2Si$						117<br>75<br>132		522539	
18	32.838	Lignoceric acid	Fatty acids	$C_{27}H_{56}O_2Si$						73<br>117<br>75		522540	
19	34.891	γ-Tocopherol	Fatty acids	$C_{31}H_{56}O_2Si$						488<br>223<br>73		91753464	
20	35.495	Hexacosanoic acid	Fatty acids	$C_{29}H_{60}O_2Si$						73<br>117<br>75		91745497	
21	36.696	α-Tocopherol	Fatty acids	$C_{32}H_{58}O_2Si$						503<br>73<br>237		14985	
22	36.849	Lanost-8-ene-3β, 7α-diol, 3-acetate	Fatty acids	$C_{32}H_{54}O_3$						468<br>75<br>57		74331694	
23	38.465	Stigmasterol	Fatty acids	$C_{32}H_{56}OSi$						83<br>129<br>55		91698162	
24	39.402	Stigmast-5-ene, 3β-(trimethylsiloxy)	Fatty acids	$C_{32}H_{58}OSi$						129<br>73<br>357		91746541	
25	43.591	Ursolic acid	Fatty acids	$C_{36}H_{64}O_3Si$						203<br>73<br>202		156700346	

Table S1: Identified low-polar metabolites by GC-MS.
